# Adherence to cancer prevention recommendations is associated with a lower breast cancer risk in black urban South African women

**DOI:** 10.1017/S0007114521001598

**Published:** 2022-03-28

**Authors:** Inarie Jacobs, Christine Taljaard-Krugell, Mariaan Wicks, Herbert Cubasch, Maureen Joffe, Ria Laubscher, Isabelle Romieu, Carine Biessy, Marc J. Gunter, Inge Huybrechts, Sabina Rinaldi

**Affiliations:** 1 Centre of Excellence for Nutrition, North-West University, Private Bag X6001, Potchefstroom 2520, South Africa; 2 Department of Surgery, Faculty of Health Sciences, University of Witwatersrand, Private Bag X2600, Houghton, Johannesburg 2041, South Africa; 3 Non-Communicable Diseases Research Division, Wits Health Consortium (PTY) Ltd, Parktown, Johannesburg 2193, South Africa; 4 MRC Developmental Pathways to Health Research Unit, Department of Paediatrics, Faculty of Health Sciences, University of Witwatersrand, Private Bag X3, Johannesburg 2050, South Africa; 5 South African Medical Research Council, PO Box 19070, Tygerberg, Cape Town 7505, South Africa; 6 Centro de Investigación en Salud Poblacional, Instituto Nacional de Salud Pública, CP 62100, Cuernavaca, Morelos, México; 7 Hubert Department of Global Health, Emory University, Atlanta, GA 30329, USA; 8 International Agency for Research on Cancer, Section of Nutrition and Metabolism, 150 cours Albert Thomas, 69372 Lyon, France

**Keywords:** Breast cancer prevention, Diet and cancer, World Cancer Research Fund/American Institute for Cancer Research recommendations, Black urban women, South Africa

## Abstract

Breast cancer prevention is of great importance to reduce high incidence in South Africa. This study aimed to investigate adherence to the 2018 World Cancer Research Fund/American Institute for Cancer Research (WCRF/AICR) Cancer Prevention Recommendations and the association with breast cancer risk in black urban women from Soweto, South Africa. A total of 396 breast cancer cases and 396 population-based controls from the South African Breast Cancer study (SABC) matched on age and demographic settings were included. Validated questionnaires were used to collect dietary and epidemiological data. To assess adherence to these recommendations, an eight-point adherence score was developed, using tertiles among controls for scoring each recommendation (0, 0·5 and 1) with zero indicating the lowest adherence to the recommendations. OR and 95 % CI were estimated using multivariate logistic regression models to analyse associations between the WCRF/AICR score and breast cancer risk. Greater adherence (>4·5 *v*. <3·25) to the 2018 WCRF/AICR Cancer Prevention Recommendations was associated with a significant inverse association with breast cancer risk overall (OR = 0·54, 95 % CI 0·35, 0·91) and specifically in postmenopausal women (OR = 0·55, 95 % CI 0·34, 0·95), in cases with oestrogen positive and progesterone positive breast cancer subtypes (OR = 0·54, 95 % CI 0·39, 0·89 and OR = 0·68, 95 % CI 0·43, 0·89, respectively) and in obese women (OR = 0·52, 95 % CI 0·35, 0·81). No significant association with breast cancer risk was observed in premenopausal women. Greater adherence to the 2018 WCRF/AICR Cancer Prevention Recommendations may reduce breast cancer risk in this black urban population of Soweto. Adherence thereof should be encouraged and form a part of cost-effective breast cancer prevention guidelines.

Breast cancer is the most commonly diagnosed cancer in women across the globe and the most common incident cancer in South African women^(1,2)^. In South Africa, breast cancer has an age-standardised rate of 49 per 100 000 women and a mortality rate of 16·3 per 100 000 in 2018^(1)^. Considering the emergence of the ongoing nutrition transition in Southern African countries, this burden is prospected to double by 2030 if no preventive actions are taken^(3,4)^. Access to early detection and treatment of breast cancer are limited and contribute to high breast cancer incidence and mortality rates in South Africa^(5)^. Primary prevention guidelines are therefore urgently required to aid in the reduction of breast cancer incidence rates and the burden on the public healthcare system^(6)^. Although policy and guidelines exist in South Africa for early detection and referral of patients with breast cancer symptoms, primary prevention guidelines have not yet been developed^(5,7)^. As an important first step in guiding the development of primary breast cancer prevention guidelines for South Africa, evidence of lifestyle (including dietary) factors in association with breast cancer risk in this population is needed^(6)^.

In 2007, experts from the World Cancer Research Fund (WCRF) and American Institute for Cancer Research (AICR) developed a public health message to promote cancer prevention in the form of ten Cancer Prevention Recommendations focusing on diet, body weight and physical activity^(8)^. Adherence to the WCRF and AICR recommendations, for instance, high consumption of fruit, vegetables and whole grains, being active and low consumption of processed meat and alcoholic beverages, reduces the risk of developing cancer, including breast cancer, and other non-communicable diseases^(9–18)^. When these Recommendations were revised in 2018, concern was drawn to the different scoring algorithms used to assess adherence to the 2007 WCRF/AICR Cancer Prevention Recommendations. Therefore, a standardised scoring algorithm (by a collaborative team from the US National Cancer Institute (NCI), AICR and WCRF International, WCRF/AICR Continuous Update Project Expert Panel and international researchers) was developed to assess adherence to the updated recommendations and to enhance comparability between studies^(18,19)^. Evidence used to compile the NCI-led standardised scoring algorithm is predominantly based on results of studies compiled in high-income countries^(19)^.

Greater adherence to the 2018 WCRF/AICR’s Cancer Prevention Recommendations, using the NCI-led standardised scoring algorithm, has shown inverse associations with breast cancer risk in high-income countries^(15)^. However, little is known about the impact of adhering to these recommendations in low-and-middle-income populations such as black urban South African women.

Promotion of adherence to the 2018 WCRF/AICR’s Cancer Prevention Recommendations may contribute in establishing affordable breast cancer prevention guidelines in South Africa. Therefore, this study aimed to investigate the association between higher adherence to the 2018 WCRF/AICR’s Cancer Prevention Recommendations and breast cancer risk in black urban women from Soweto, South Africa. Adherence to individual recommendations of the 2018 WCRF/AICR Cancer Prevention Recommendations will also be investigated.

## Materials and methods

The database from the South African Breast Cancer (SABC) study, a population-based, case–control study (breast cancer cases *n* 396, controls *n* 396) conducted among black urban women from the greater Soweto population, in South Africa, was used to conduct this study^(20,21)^. Breast cancer case participants (between the ages 27 and 86 years) were newly diagnosed breast cancer patients from the Chris Hani Baragwanath Academic hospital in Soweto, Gauteng, who were enrolled in the study as soon as possible after their cancer diagnoses before receiving any cancer treatments. The stage at breast cancer was clinically assessed at diagnoses and coded according to the tumour-node-metastasis classification. Controls (between the ages 26 and 88 years) were healthy (no illness or being pregnant) and unrelated to breast cancer cases with no history of cancer diagnoses and matched by age (±5 years) and area of residence. Participants were recruited from December 2014 to June 2017. The sample size had a sufficient power of 80 % (when type II error rate = 10 %) for OR ≥ 1·5 and type I error set at 5 %^(22)^.

### Ethical approval

The International Agency for Research on Cancer and the University of the Witwatersrand Committee for Research on Human Subjects granted ethical approval for the South African Breast Cancer study (M140980). Permission to conduct research at Chris Hani Baragwanath academic hospital was obtained from the Gauteng Province Medical Advisory Committee. All subjects gave written informed consent prior to participation.

### Determining habitual dietary intake

A reproducible and validated culture-specific quantified FFQ (QFFQ) (Spearman rank correlation coefficients of 0·14–0·59 for validation/reproducibility when macronutrient and micronutrient intakes were compared with a 7-d weighed food record and foods captured by a QFFQ) was used together with food portion pictures, food models and household utensils^(23–25)^. The QFFQ consisted of 145 food items reported by recently published literature as frequently consumed staple foods and foods less frequently consumed. The dietary intake frequency included the amount of times foods were consumed per d/week per month or never. Life-size colour photographs of thirty-seven foods (in three portion sizes) were displayed in the food portion picture booklet. Participants were asked about their habitual dietary intake over the past month^(25)^. Daily intakes of the different foods included in the QFFQ were calculated via a stepwise approach. At first, consumption frequencies were converted into number of days per month. Then, the amount of each serving consumed (for each individual) was converted into g using standardised tables to convert household measurements into g^(26)^. Finally, the daily consumption was calculated by multiplying the frequencies of consumption (d/month) with the portion sizes divided over 30 d. The daily energy and nutrient intakes were determined by multiplying the daily intake of each food item (as consumed) by the nutrient and energy content (per 100 g), derived from the South African Food Composition tables, and then adding the contribution from all food items together^(26)^.

### Non-dietary assessments

Trained fieldworkers and investigators conducted face-to-face interviews. Detailed information on demographic and socio-economic status such as ethnicity, history of health, family history of breast cancer, reproductive risk factors (age/year at full-term pregnancy, breast-feeding history for each live birth, age at menarche and at menopause for postmenopausal women, use of oral contraceptives and hormone replacement therapy), family history of cancer, breast health (previous breast lumps by breast laterality and breast pains), smoking habits and physical activity (recreational and household) was collected. Weight, height, sitting height and waist circumference were measured according to Lohman’s laws^(27)^. All questionnaires used to obtain the anthropometry and lifestyle information were validated and proven reproducible in studies conducted in South Africa and elsewhere^(28–30)^.

### Construction of the World Cancer Research Fund/American Institute for Cancer Research Cancer Prevention Recommendations adherence score

The SABC study database was used to calculate an adherence score to the 2018 WCRF/AICR’s Cancer Prevention Recommendations and to assess adherence to individual recommendations of the 2018 WCRF/AICR’s Cancer Prevention Recommendations^(19)^. Table 1 provides details regarding the inclusion criteria and food items that were included in each food group^(18,19)^.

**Table 1.** Inclusion criteria and items included in specific groups of the 2018 World Cancer Research Fund/American Institute for Cancer Research Cancer Prevention Recommendations

Recommendation group	Inclusion criteria	Items included
Non-starchy vegetables	Relatively unprocessed non-starchy vegetables.	Green leafy vegetables, broccoli, okra, onions, tomatoes, aborigine, cauliflower, pumpkin, cabbage, carrots and beetroot.
Fruits	Relatively unprocessed fruits.	Apples, pears, bananas, oranges, grapes, nectarines, peaches, watermelon, mango, all berries, avocados, plums and pineapple.
Fast food, and processed foods high in fat, refined starches or sugar	Foods in this food group were classified using the NOVA classification system, calculated as a percentage of energy (kJ) from ultraprocessed foods (solid, semi-solid and liquid foods) divided by the total energy intake/d (kJ) for each participant^(46)^.	Fast foods, pre-prepared dishes, snacks, bakery foods and confectionery high in fat, refined starches or added sugars such as pizza, potato fries, hamburgers, deep fried foods, candies and desserts. Sugar-sweetened beverages, alcoholic beverages and processed meat were excluded to avoid double penalisation.
Red meat	All mammalian muscle meat. Organ and offal meat were included in the analysis since consumption thereof is higher in this population compared with other populations.	All beef, veal, pork, mutton, lamb, horse and goat meat (not processed).
Processed meat	All meat that has been altered by the processes of curing, salting, fermentation, smoking and other methods used to enhance flavour or improve preservation.	Sausages, polonies, meat loafs, Russians, corned beef, Vienna’s
Sugar-sweetened beverages	Liquids with added free sugars (sucrose, fructose, maize syrup and sugars naturally present in honey, syrups, fruit juices and fruit juice concentrates), sugar-free and artificially sweetened drinks.	Carbonated soft drinks (Coca-Cola products etc.), dairy and fruit juice blends, cordials, fruit juice with added sugar and energy drinks.
Breast-feeding	The duration of cumulative breast-feeding in case and control participants (separately) was used to determine adherence to the breast-feeding guideline.	Breast-feeding of 6 months and above.
*Alcoholic beverages	Any beverage containing alcohol and home-made beer were included.	Beer, wine, ciders and spirits.

* Amount of ethanol in alcoholic beverages (adapted from Shams-White *et al.*, 2019)^(19)^

Each of the participants’ adherence to the 2018 WCRF/AICR Cancer Prevention Recommendations was calculated for overall adherence as well as each individual recommendation of the 2018 WCRF/AICR Cancer Prevention Recommendations. Adherence points were calculated as follows: one (1) point when adhering to the recommendation; 0·5 points for partial adherence and lastly, zero (0) points for non-adherence to the recommendation. Recommendations regarding cancer survivors and using supplementation to prevent cancer were not applicable to this study and were therefore excluded from the adherence score. A maximum overall adherence score of eight was therefore possible, where zero (0) indicates the lowest and eight (8) the highest adherence to the overall score. Each individual recommendation contributed equally to the total adherence score. The recommendations, ‘be a healthy weight’ and ‘eat wholegrains, fruit, vegetables and beans’, had two sub-recommendations that were scored individually and divided by two to determine an average score (0, 0·25 and 0·5). The recommendation on breast-feeding was only applied to parous women (controls = 96·2 %, cases = 95·6 %).

Adherence distributions to some of the individual WCRF/AICR’s recommendations (meeting, partially meeting or not meeting the recommendation) were highly skewed (online Supplementary Table S1). Thus, using the predefined NCI cut-offs may result in insufficient statistical power to assess adherence to the 2018 WCRF/AICR Cancer Prevention Recommendations and breast cancer risk in our study due to this (rather overweight and obese) population group. Therefore, the authors decided to use an alternative approach to score the individuals (0, 0·5 and 1) for their level of adherence to the 2018 WCRF/AICR Cancer Prevention Recommendations by using data-driven tertiles derived from the controls (33rd and 66th percentiles). Recommendations based on the data-driven tertiles in the current study population will be referred to as ‘adapted WCRF/AICR Cancer Prevention Recommendations’ (see Table 2). Recommendations based on the original NCI-led standardised scoring algorithm will be referred to as the 2018 WCRF/AICR Cancer Prevention Recommendations.

**Table 2.** Distribution of adherence to the individual recommendations of the adapted World Cancer Research Fund (WCRF)/American Institute for Cancer Research (AICR) Cancer Prevention Recommendations between breast cancer cases and controls, using population-based tertiles (33rd and 66th percentiles) (Numbers and percentages)

2018 WCRF/AICR cancer prevention recommendation	Operationalisation	Score contribution	Controls (*n* 396)		Cases (*n* 396)		*P*
			*n*	%	*n*	%	
1. Be a healthy weight*	BMI: ≤ 28·6 kg/m^2^	0·5	131	33·1	143	36·5	
	28·6 kg/m^2^ < BMI < 34·7 kg/m^2^	0·25	131	33·1	125	31·9	0·669
	BMI: >34·7 kg/m^2^	0	134	33·8	128	31·6	
2. Be a healthy weight*	Waist circumference: ≤ 92 cm	0·5	139	35·1	167	42·2	
	92 cm < Waist circumference <102 cm	0·25	130	32·8	130	32·8	0·049
	Waist circumference: ≥ 102 cm	0	127	32·1	99	25·0	
2. Be physically active	Moderate or vigorous PA ≥ 51 min/week	1	150	37·9	173	44	
	20 min/week < Moderate or vigorous PA < 51 min/week	0·5	97	24·5	65	16·4	0·100
	Moderate or vigorous PA ≤ 20 min/week	0	149	37·6	158	39·9	
3. Eat a diet rich in wholegrains, vegetables, fruit and beans†	Fruits and vegetables ≥ 543 g/d	0·5	131	33·1	152	38·4	
	169 g ≤ Fruits and vegetables <543 g/d	0·25	134	33·8	85	21·5	<0·001
	Fruits and vegetables <169 g/d	0	131	33·1	159	40·2	
3. Eat a diet rich in wholegrains, vegetables, fruit and beans†	Dietary fibre ≥ 27·1 g/d	0·5	152	38·4	147	37·1	
	19·1 g ≤ Dietary fibre <27·1 g/d	0·25	92	23·2	103	26·0	0·955
	Dietary fibre <19·1 g/d	0	152	38·4	146	36·9	
4. Limit consumption of ‘fast foods’ and other processed foods high in fat, refined starches or sugars	Total kJ from UPF ≤ 20·1 % of TEI	1	133	33·6	152	38·4	
	20·1 % <Total kJ from UPF ≥ 50·1 % of TEI	0·5	129	32·6	156	39·4	0·001
	Total kJ from UPF >50·1 % of TEI	0	134	33·8	88	22·2	
5. Limit consumption of red and processed meat	Red meat ≤ 60 g/week and processed meat ≤ 8·6 g/d	1	58	14·6	92	23·2	
	60 g/week < Red meat and ≤ 317 g/week and 8·6 g/d < processed meat and ≤ 51·4 g/d	0·5	236	59·6	247	62·4	<0·001
	Red meat > 317 g/week and processed meat > 51·4 g/d	0	102	25·8	57	14·4	
6. Limit consumption of sugar-sweetened drinks	Sugary drinks: ≤59 g/d	1	131	33·1	133	33·6	
	59 g/d < Sugary drinks < 200 g/d	0·5	141	35	147	37·1	0·521
	Sugary drinks ≥ 200 g/d	0	124	31	116	29·3	
7. Limit alcohol consumption	Ethanol intake 0 g/d	1	321	81·1	350	88·4	
	0 g/d < Ethanol intake ≤ 4·6 g/d	0·5	41	10·4	25	6·3	0·017
	Ethanol intake > 4·6 g/d	0	34	8·5	21	5·3	
8. Do not rely on supplements for cancer prevention	Not applicable to this population						
9. For mothers: breast-feed your baby, if you can‡	Cumulative breast-feeding ≥ 48 months	1	120	31·8	138	36·1	
	22 months < Cumulative breast-feeding < 48 months	0·5	118	31·3	117	30·6	0·761
	Cumulative breast-feeding ≤ 22 months	0	139	36·9	127	33·3	
10. After a cancer diagnosis: follow our recommendations, if you can	Not applicable to this population						

PA, physical activity; UPF, ultraprocessed food.

* Adherence to the recommendation ‘Be a healthy weight’ is measured by two subgroups, BMI and waist circumference.

† Adherence to the recommendation ‘Eat a diet rich in wholegrains, vegetables, fruit and beans’ is measured by two subgroups, fruit and vegetable intake and daily fibre intake.

‡ Recommendation only applied to parous women (case *n* 382; controls *n* 377).

### Statistical analysis

A total of 399 breast cancer cases and 399 controls were recruited in the SABC study. Of those, three breast cancer cases and three matched controls were excluded due to missing dietary information. Descriptive analyses were performed, and differences between breast cancer cases and controls were assessed using a paired sample *t* test (normally distributed data presented as mean values and standard deviation) and Wilcoxon Signed-Rank test for skewed data with a skewness value <−0·5 and >0·5 (presented as median, 25th and 75th percentiles) for continuous variables. Paired *χ* ^2^ test was used for categorical variables (presented as percentage of breast cancer cases or controls, respectively, within a category). Specifications of the WHO were used to calculate BMI, using measured height and weight (kg/m^2^).

### Assessing adherence to the World Cancer Research Fund/American Institute for Cancer Research Cancer Prevention Recommendations: overall and individual recommendations

To determine the association between breast cancer risk and adherence to both the 2018 WCRF/AICR and adapted WCRF/AICR Cancer Prevention Recommendations (overall and individual recommendations), conditional logistic regression models were used to compute OR and associated 95 % CI. Adherence scores (overall and for each individual recommendation) were stratified by hormonal breast cancer receptor subtypes (ER+ and PR+), menopausal status and obesity. Since breast cancer cases and controls were not matched on menopausal or obesity status, unconditional logistic regression was used when adherence (overall and for each individual recommendation) analyses were stratified by menopausal status (pre *v*. post) and obesity (limited to BMI ≥ 30 kg/m^2^ due to sample size).

The following confounders were examined for adherence to the overall and individual recommendations of the 2018 WCRF/AICR Cancer Prevention Recommendations (as well as the adapted WCRF/AICR Cancer Prevention Recommendations): ethnicity (Zulu/Pedi/Swazi, Xhosa, Sotho, Tshwane, Venda, Tsonga and Ndebele), individual income (R1–R3000, R3001–R6000 and R6001+), level of education (none/primary school, high school and college/postgraduate/diploma), smoking (smokers and non-smokers), height (highest *v*. lowest), age at menarche (continuous), full-term pregnancy (yes/no), age at first pregnancy (<24 *v.* >24 years of age), age at menopause (<48 *v.* >48 years of age), parity (≤three children *v.* >three children), duration of exclusive breast-feeding (months), use of exogenous hormones (hormonal birth control to avoid pregnancy: oral contraceptives and injections), or hormone replacement therapy after menopause), family history of breast cancer (yes/no), HIV status (positive *v*. negative), total energy intake in kJ (continuous) and under-reporting (under-reporting, plausible reporting and over-reporting). Under-reporting (13·1 % of breast cancer cases and 11·6 % of controls) and over-reporting (24·0 % of breast cancer cases and 27 % of controls) cut-off points were calculated using the Goldberg and Black principle to determine over- and under-reporting of energy intake^(31,32)^. The Goldberg and Black principle is based on the following idea: if weight is stable, then energy expenditure equals energy intake^(31,32)^.

Only menopausal status, ethnicity, total energy intake, income and level of education altered the crude OR by more than 10 % when assessing adherence to overall and individual recommendations and were included in our final model.

Additional confounders were examined when adherence to the individual recommendations of both 2018 WCRF/AICR Cancer Prevention Recommendations and adapted WCRF/AICR Cancer Prevention Recommendations was assessed. This was done as the following confounders were part of the overall score and as such not included as confounders in the overall score analyses: waist circumference (continuous data), habitual physical activity per d (active and less active), ever breast-feeding (yes/no) and alcohol consumption (continuous). These additional confounders were only included in the analysis when not part of the individual recommendation itself.

Although this study mainly focuses on the results of scoring systems based on data-driven analysis of the adapted 2018 WCRF/AICR Cancer Prevention Recommendations (Table 2), sensitivity analyses were run to compare our results with the adherence results (overall and individual recommendations), using the NCI-led standardised scoring algorithm (online Supplementary Tables S2 and S3). These analyses were run as sensitivity analyses because predefined cut-points used in the NCI-led standardised scoring algorithm in our population (2018 WCRF/AICR Cancer Prevention Recommendations) resulted in highly skewed adherence categories (meeting, partially meeting or not meeting) of the 2018 WCRF/AICR Cancer Prevention Recommendations.

## Results

Table 2 presents the distribution of adherence to individual recommendations of the adapted WCRF/AICR’s Cancer Prevention Recommendations between breast cancer cases and controls. Selected characteristics amongst breast cancer case and control participants are reported in Table 3. Ethnicity differed significantly between cases and control participants with cases having more Ndebele-speaking people and controls having more Sotho-speaking people. Breast cancer cases had a significant lower waist circumference (93·3 (sd 13·8) cm) compared with controls (95·8 (sd 13·7) cm) and were often less HIV-positive (16·5 %) than controls (22·6 %). Controls had higher percentage of alcohol drinkers, higher consumption of ethanol (g/d), higher intakes of fruit, non-starchy vegetables, red-and-processed meat and energy-dense, fast foods than cases. Hormone-responsive breast cancers, ER+ (75·2 %) and PR+ (75·2 %) were the dominant breast cancer subtypes, while triple-negative breast cancer accounted for 16·1 % (not stratified by menopausal status).

**Table 3.** Distribution of characteristics between breast cancer case and control participants (continuous variables are presented as means and standard deviations if normally distributed and median (25th percentile, 75th percentile) if not, categorical variables are presented as percentages)

Characteristics	Controls (*n* 396)		Cases (*n* 396)		*P*
	*n*	%	*n*	%	
Socio-demographic factors					
Age (years)					0·997
Mean	54·6		54·7		
sd	12·9		12·9		
Ethnicity					0·004
Zulu and Pedi	25	6·4	26	6·5	
Xhosa	22	5·6	22	5·5	
Sotho	144	36·4	108	27·4	
Tswana	19	4·8	19	4·8	
Venda	56	14·1	40	10·1	
Tsonga	35	8·8	65	16·4	
Ndebele	95	23·9	116	29·3	
Level of education					0·078
None/primary	71	18·0	97	24·5	
High school	279	70·5	257	64·9	
College/University/postgraduate	46	11·6	42	10·6	
Individual income/month					0·350
R0	108	27·3	125	31·6	
R1–R3000	227	57·3	219	55·3	
R3001–R6000+	61	15·4	52	13·1	
Anthropometry					
BMI (kg/m^2^)					0·317
Mean	31·8		31·4		
sd	6·9		7·0		
WC (cm)					0·016
Mean	95·8		93·3		
SD	13·7		13·8		
Lifestyle factors					
PA (MET/week)	110·2	81·6; 149·7	114·0	82·8; 163·1	0·197
Total vigorous and moderate PA min/week	32·06	9·1; 70·8	39·4	7·8; 85·8	0·303
Current smokers	44	11·1	35	8·8	0·286
HIV positivity	90	22·6	65	16·5	0·025
Dietary factors					
TE (kJ/d)	8990	7184; 10 284	9146	6812; 9759	0·239
Protein (g/d)	63·5	49·2; 93·1	63·8	47·4; 82·7	0·073
% of TE	12·0		11·8		
Fat (g/d)	64·4	47·2; 95·7	64·8	42·4; 91·9	0·125
% of TE	27·2		26·9		
CHO (g/d)					0·417
Mean	338·7		330·8		
sd	147·3		143·5		
% of TE	64·0		61·4		
Dietary fibre (g/d)					0·629
Mean	25·3		24·9		
sd	11·4		11·03		
Added sugar (g/d)	67·9	39·9; 109·7	65·3	38·4; 105·5	0·313
% of TE	12·0		12·1		
Energy from alcohol (kJ/d)	312	288; 2204	79	29; 1954	0·382
% alcohol contribution to TE	3·4		0·8		
Alcohol non-consumers	321	81·1	350	88·4	0·004
Ethanol intake (g/d)	4·6	2·5; 14·7	5·4	2·8; 13·8	0·005
Fruit and non-starchy vegetables (g/d)	296·6	126·9; 700·1	240·2	108·3; 483·5	0·001
% Energy (kJ) from fast foods to TEI/d (%)*	26·9	16·8; 56·8	24·7	15·6; 37·7	0·004
Red meat intake (g/week)	142·2	35·0; 485·0	55·8	22·0.; 157·5	<0·001
Processed meat intake (g/d)	18·5	4·7; 100·01	12·9	2·5; 42·8	0·004
Sugary drinks (g/d)	100·0	17·8, 285·7	100·0	19·6; 257·1	0·576
Breast cancer risk factors					
Ever pregnant (n/%)	382	96·5	377	95·2	0·374
Ever breast-fed in parous women (n/%)	349	91·4	339	89·9	0·496
Duration of breast-feeding (months)*	41	24; 62	35	20; 62	0·259
Use of birth control (contraceptives)	215	54,3	229	57·8	0·316
†Postmenopausal	257	64·9	248	62·6	0·852
Age at menarche	15	13; 16	15	13; 16	0·251
Age at menopause‡ (years)	48	44; 50	47	42; 5	0·792
Family history of breast cancer	17	4·3	25	6·3	0·205
Breast cancer case characteristics					
Stage at BC diagnoses					
I			24	6·5	
II			175	44·8	
III			161	40·8	
IV			31	7·9	
Receptor status					
ER+			298	75·2	
PR+			263	66·4	
HER2			114	28·8	
Breast cancer case subtype					
HER2 enriched			21	5·3	
Luminal A			40	10·1	
Luminal B			269	67·9	
TNBC			64	16·1	

WC, waist circumference; TE, total energy; CHO, carbohydrates; PA, physical activity; ER+, oestrogen receptor positive; PR+ progesterone receptor positive; HER2, Human-Epidermal Growth Factor-2; TNBC, Triple negative breast cancer; HRT, hormone replacement therapy.

* In breast-feeding women only.

† 20 missing values for menopausal status (15 cases and 5 control). Missing values were excluded from percentage calculations.

‡ Among postmenopausal women only.

Adherence to the overall adapted WCRF/AICR’s Cancer Prevention Recommendations is presented in Table 4 together with breast cancer risk associations. Overall, only 13·9 % of controls and 19·7 % of cases adhered to more than half (>4·5 out of 8) of the overall adapted WCRF/AICR Cancer Prevention Recommendations in this population. Highest adherence (4·5–8) *v*. lowest adherence (0–3·25) to the adapted WCRF/AICR Cancer Prevention Recommendations showed a significant inverse association with breast cancer risk overall (OR = 0·54, 95 % CI 0·35, 0·91, *P* = 0·009) as well as in postmenopausal women (OR = 0·55, 95 % CI 0·34, 0·95, *P* = 0·032), in ER+, in PR+ breast cancer’s (OR = 0·54, 95 % CI 0·39, 0·89, *P* = 0·019 and OR = 0·68, 95 % CI 0·43, 0·89, *P* = 0·029, respectively) and in obese women (OR = 0·52, 95 % CI 0·35, 0·81, *P* = 0·017). Furthermore, per one-point increment adherence to the adapted WCRF/AICRS’s Cancer Prevention Recommendations showed an inverse association with breast cancer risk overall (OR = 0·86, 95 % CI 0·73, 0·98, *P* = 0·019) and in participants with ER+ and PR+ breast cancers (OR = 0·87, 95 % CI 0·79, 0·92, *P* = 0·036 and OR = 0·69, 95 % CI 0·53, 0·91, *P* = 0·008, respectively). No significant association with breast cancer risk was observed in premenopausal women (overall and per one-point increment adherence). Sensitivity analyses were conducted by excluding under- and over-reporters (according to the Goldberg and Black principle)^(31,32)^ and HIV-positive cases in the analysis (separately) but did not alter the outcome (results not shown).

**Table 4:** Associations between the adherence to the overall adapted World Cancer Research Fund (WCRF)/American Institute for Cancer Research (AICR) Cancer Prevention Recommendation using data-driven cut-points (33rd and 66th percentiles) and breast cancer risk (Numbers and percentages)

	Adherence score	Controls		Cases					
		*n*	%	*n*	*%*	Model 1		Adjusted Model 2	
Overall breast cancer (cases *n* 396; controls *n* 396)	Per 1-point increment					0·92	0·81–1·01	0·86	0·73–0·98
	*P*					0·067		0·019	
	≤3·25	215	54·3	191	48·2	1		1	
	>3. 25 and ≤4·5	126	31·8	127	32·1	0·88	0·65–1·23	0·82	0·57–1·18
	>4·5	55	13·9	78	19·7	0·64	0·43–0·95	0·54	0·35–0·91
	*P*_trend_					0·027		0·009	
Premenopausal*,† (*n* 267) (cases *n* 133; controls *n* 134)	Per 1-point increment					0·80	0·63–1·14	0·87	0·70–1·19
	*P*					0·345		0·121	
	≤3·25	61	45·5	69	51·9	1		1	
	>3. 25 and ≤4·5	43	32·1	45	33·8	0·97	0·56–1·63	0·91	0·53–1·68
	>4·5	30	22·4	19	14·3	0·57	0·33–1·1	0·56	0·28–1·19
	*P*_trend_					0·093		0·088	
Postmenopausal*,† (*n* 505) (cases *n* 248; controls *n* 257)	Per 1-point increment					0·91	0·72–1·014	0·86	0·74–1·08
	*P*					0·132		0·112	
	≤3·25	128	49·8	139	56·0	1		1	
	>3 25 and ≤4·5	82	31·9	77	31·0	0·87	0·54–1·33	0·86	0·53–1·38
	>4·5	47	18·3	32	13·0	0·60	0·37–1·04	0·55	0·34–0·95
	*P*_trend_					0·075		0·032	
ER+ (*n* 298)	Per 1-point increment					0·72	0·65–1·03	0·87	0·79–0·92
	*P*					0·075		0·036	
	≤3·25			157	52·7	1		1	
	>3 25 and ≤4·5			93	31·2	0·83	0·57–1·62	0·79	0·52–1·18
	>4·5			48	16·1	0·47	0·19–1·01	0·54	0·39–0·89
	*P*_trend_					0·054		0·019	
PR+ (*n* 263)	Per 1-point increment					0·59	0·54–0·81	0·69	0·53–0·91
	*P*					0·005		0·008	
	≤ 3·25			134	51·0	1		1	
	>3 25 and ≤4·5			89	33·8	0·80	0·41–1·21	0·60	0·31–1·17
	>4·5			40	15·2	0·42	0·17–0·75	0·68	0·43–0·89
	*P*_trend_					0·012		0·029	
Obese (*n* 466) Cases = 231 Controls = 235*,‡	Per 1-point increment					0·88	0·73; 0·98	0·82	0·61; 0·96
	*P*					0·020		0·234	
	≤ 3·25	149	63·5	168	72·7	1		1	
	>3 25 and ≤ 4·5	59	25·1	48	20·8	0·80	0·53–1·23	0·74	0·47–1·07
	>4·5	27	11·4	15	6·5	0·61	0·43–0·98	0·52	0·35–0·81
	*P*_trend_					0·039		0·017	

ER+, oestrogen receptor positive; PR+, progesterone receptor positive

Model 1: crude model.

Adjusted model 2: Adjusted for total energy intake, ethnicity, level of education, level of income and menopausal status (not adjusted for menopausal status when stratified by menopausal status).

* Unconditional logistic regression.

† 20 missing values for menopausal status (15 cases and 5 controls).

‡ Obesity defined as BM I ≥ 30 kg/m^2^.

Adherence to individual recommendations of the adapted WCRF/AICR Cancer Prevention Recommendations, using data-driven tertiles, is presented in Table 5. After adjusting for possible confounding factors, smaller waist circumferences showed a positive association with breast cancer risk overall (OR = 1·6, 95 % CI 1·13, 2·46, *P* = 0·010), in postmenopausal women (OR = 1·69, 95 % CI 1·08, 2·63, *P* = 0·020), in participants with ER+ breast cancers (OR = 1·46, 95 % CI 1·00, 2·13, *P* = 0·050) and in obese women (OR = 2·85, 95 % CI 1·51, 5·40, *P* = 0·001). Higher consumption of fresh fruit and non-starchy vegetables showed an inverse association with breast cancer risk overall (OR = 0·48, 95 % CI 0·32, 0·73, *P* = <0·001) and in postmenopausal women (OR = 0·55, 95 % CI 0·34, 0·88, *P* = 0·014), while lower fast-food consumption showed an inverse association with breast cancer risk overall (OR = 0·64, 95 % CI 0·38, 0·92, *P* = 0·029). Greater adherence to the recommendation on ‘limiting alcohol consumption’ showed a positive association with premenopausal breast cancer risk (OR = 2·9, 95 % CI 1·24, 6·89, *P* = 0·014).

**Table 5.** Associations between the adherence to individual recommendations of the adapted World Cancer Research Fund (WCRF)/American Institute for Cancer Research (AICR) Cancer Prevention Recommendations using data-driven categories (33rd and 66th percentiles) and breast cancer risk (Odds ratio and 95 % confidence intervals)

WCRF/AICR cancer prevention recommendation	Adherence score	Overall* (cases *n* 396; controls *n* 396)		Premenopausal†,‡ (cases = 133; controls = 134)		Postmenopausal†,‡ (cases = 248; controls = 257)		ER† + (*n* 298)		PR† + (*n* 263)		Obese†,§ (cases = 231; controls = 235)	
		OR	95 % CI	OR	95 % CI	OR	95 % CI	OR	95 % CI	OR	95 % CI	OR	95 % CI
Be a healthy weight|| BMI	0	1		1		1		1		1		1	
	0·5	1·02	0·70, 1·48	1·47	0·78, 2·75	0·83	0·54, 1·28	0·95	0·65, 1·38	0·91	0·54, 1·54	0·84	0·72, 1·10
	1	1·09	0·76, 1·57	1·01	0·56, 1·81	1·18	0·76, 1·83	1·09	0·75, 1·58	0·84	0·49, 1·43	1·12	0·76, 1·64
	*P*_trend_	0·622		0·978		0·454		0·635		0·519		0·553	
Be a healthy weight|| Waist circumference	0	1		1		1		1		1		1	
	0·5	1·3	0·89, 1·92	1·37	0·69, 2·72	1·25	0·81, 1·93	1·20	0·81, 1·77	1·19	0·68, 2·08	1·28	0·86, 1·92
	1	1·6	1·13, 2·46	1·30	0·69, 2·44	1·69	1·08, 2·63	1·46	1·00, 2·13	1·43	0·83, 2·46	2·85	1·51, 5·40
	*P*_trend_	0·010		0·406		0·020		0·050		0·200		0·001	
Be physically active	0	1		1		1		1		1		1	
	0·5	0·77	0·53, 1·14	1·41	0·74, 2·69	0·60	0·38, 0·93	0·77	0·51, 1·18	0·43	0·19, 0·98	0·76	0·47, 1·23
	1	1·15	0·81, 1·64	1·52	0·86, 2·68	1·07	0·69, 1·65	1·29	0·87, 1·92	0·71	0·33, 1·48	1·20	0·75, 1·93
	*P*_trend_	0·420		0·149		0·751		0·195		0·363		0·446	
Eat a diet rich in wholegrains, vegetables, fruit and beans¶ Fruit and vegetable intake	0	1		1		1		1		1		1	
	0·5	0·97	0·68, 1·37	0·77	0·44, 1·37	1·25	0·81, 1·93	0·77	0·51, 1·18	1·55	0·76, 3·17	0·99	0·64, 1·55
	1	0·48	0·32, 0·73	0·63	0·33, 1·20	0·55	0·34, 0·88	1·29	0·87, 1·92	0·61	0·26, 1·45	0·62	0·38, 1·02
	*P*_trend_	<0·001		0·162		0·014		0·195		0·264		0·124	
Eat a diet rich in wholegrains, vegetables, fruit and beans¶ Daily fibre intake	0	1		1		1		1		1		1	
	0·5	1·02	0·69, 1·51	0·87	0·42, 1·79	1·04	0·67, 1·62	1·03	0·66, 1·57	0·87	0·38, 2·07	1·32	0·81, 2·16
	1	0·93	0·59, 1·47	0·65	0·29, 1·44	1·18	0·69, 2·02	0·91	0·67, 1·51	0·99	0·36, 2·65	1·09	0·61, 1·94
	*P*_trend_	0·760		0·295		0·541		0·709		0·986		0·766	
Limit fast foods	0	1		1		1		1		1		1	
	0·5	0·84	0·51, 1·11	0·80	0·45, 1·79	0·97	0·68, 1·49	0·79	0·36, 1·71	0·64	0·49, 1·55	0·98	0·62, 1·63
	1	0·64	0·38, 0·92	0·65	0·44, 1·1	0·64	0·41, 1·09	0·49	0·17, 1·13	0·53	0·33, 1·45	0·69	0·44, 1·23
	*P*_trend_	0·029		0·092		0·099		0·081		0·221		0·171	
Limit red and processed meat consumption	0	1		1		1		1		1		1	
	0·5	0·91	0·33, 1·12	0·92	0·47, 1·79	0·43	0·27, 1·29	0·80	0·53, 1·22	0·72	0·22, 1·06	0·69	0·23, 1·04
	1	0·69	0·31, 1·06	0·54	0·26, 1·15	0·58	0·49, 1·39	0·71	0·44, 1·13	0·49	0·37, 1·00	0·47	0·26, 0·87
	*P*_trend_	0·128		0·112		0·421		0·313		0·071		0·155	
Limit sugary drink intake	0	1		1		1		1		1		1	
	0·5	1·25	0·86, 1·83	0·90	051, 1·61	1·43	0·90, 2·27	1·35	0·88, 2·07	0·92	0·43, 1·97	1·37	0·86, 2·18
	1	1·06	0·72, 1·56	1·34	0·69, 2·59	1·11	0·72, 1·720	1·07	0·68, 1·67	0·94	0·43, 2·02	0·67	0·41, 1·09
	*P*_trend_	0·751		0·375		0·652		0·758		0·869		0·110	
Limit alcohol consumption	0	1		1		1		1		1		1	
	0·5	0·65	0·35, 1·22	1·21	0·43, 3·40	0·57	0·27, 1·22	0·56	0·28, 1·12	0·27	0·68, 1·12	0·81	0·36, 1·86
	1	1·37	0·87, 2·16	2·91	1·24, 6·89	1·20	0·70, 2·06	1·17	0·71, 1·95	1·52	0·61, 3·76	1·52	0·82, 2·83
	*P*_trend_	0·216		0·014		0·507		0·532		0·366		0·181	
Breastfeed your baby, if you can	0	1		1		1		1		1		1	
	0·5	0·89	0·58, 1·21	0·74	0·40, 1·35	1·01	0·64, 1·59	0·39	0·17, 0·96	0·67	0·33, 1·38	0·89	0·57, 1·42
	1	0·83	0·47, 1·01	0·85	0·47, 1·54	0·70	0·54, 1·33	1·44	0·58, 3·62	0·86	0·41, 1·84	0·85	0·53, 1·34
	*P*_trend_	0·316		0·600		0·490		0·428		0·709		0·485	

ER+, oestrogen receptor positive; PR+, Progesterone receptor positive.

* Adjusted for total energy intake, individual income/month, ethnicity, level of education, physical activity, waist circumference, alcohol intake, ever breast-feeding (unless the variable was part of the recommendation under investigation) and menopausal status (not when stratified by menopausal status)

† Stratified by menopausal status or obesity and using unconditional logistic regression.

‡ 20 missing values for menopausal status (15 cases and 5 controls).

§ Obesity defined as BMI ≥ 30 kg/m^2^

|| Adherence to the recommendation ‘Be a healthy weight’ is measured by two subgroups, BMI and waist circumference.

¶ Adherence to the recommendation ‘Eat a diet rich in wholegrains, vegetables, fruit and beans’ is measured by two subgroups, fruit and vegetable intake and daily fibre intake.

In contrast, when looking at the adherence to individual recommendations of the 2018 WCRF/AICR Cancer Prevention Recommendations (online Supplementary Table S1), only 42 % of controls and 31 % of breast cancer cases consumed more than 400 g of fruit and non-starchy vegetables per day. As for adherence to the recommendation on limiting consumption of fast foods and other processed foods high in fat, refined starches or sugars, only 43 % of cases and 37 % of controls adhered to this recommendation (<20·1 % of total energy intake/d). Furthermore, over 80 % of both breast cancer case and control participants did not adhere to the recommendation on ‘being a healthy weight’ (BMI < 25 kg/m^2^), while only 5 % of control and 6 % of case participants adhered to the recommendation on ‘be physically active (>150 min of physical activity per week)’.

## Discussion

In this population of black urban women from Soweto, South Africa, greater adherence to the adapted WCRF/AICR Cancer Prevention Recommendations was inversely associated with the risk of developing breast cancer overall, in ER+ and PR+ cancers, as well as in postmenopausal-and-obese women. Both breast cancer cases and controls showed low adherence to the overall adapted WCRF/AICR Cancer Prevention Recommendations. As for adherence to individual recommendations of the adapted WCRF/AICR Cancer Prevention Recommendations, an inverse association with breast cancer risk was obtained with greater adherence on limiting fast-food intakes (overall) and increasing fresh fruit and vegetable intakes (premenopausal and PR+ breast cancer subtypes).

Studies investigating the relationship between adherence to the 2018 WCRF/AICR Cancer Prevention Recommendations and breast cancer risk are still scarce, especially in non-Caucasian populations. One of the first case–control studies (conducted in Italy and Switzerland) investigating this relationship showed a significant inverse association with breast cancer risk (highest *v*. lowest adherence) overall (OR = 0·60, 95 % CI 0·51, 0·70) and per one-point increment adherence to the recommendations (OR = 0·83, 95 % CI 0·65, 0·82)^(15)^. Similar results were observed in a cohort study that was conducted in two Swedish populations, showing that higher adherence to the 2018 WCRF/AICR Cancer Prevention Recommendations was inversely associated with overall cancer risk (OR = 0·88, 95 % CI 0·82, 0·95) and per one-point increment adherence to the score (OR = 0·97, 95 % CI 0·95, 0·99)^(33)^. These studies did not differentiate between menopausal status, breast cancer receptor subtypes and obesity. The overall results from this study, using an adapted scoring algorithm, are similar to these findings mentioned above, even though there was a significant difference in the population under study.

When adherence to individual recommendations of the 2018 WCRF/AICR Cancer Prevention Recommendations (using the NCI-led predefined cut-off points) was first analysed, highly skewed distributions (≥65 % of breast cancer cases and controls not meeting an individual WCRF/AICR Cancer Prevention Recommendation) were observed in the majority of the individual recommendations. For instance, over 80 % of both breast cancer case and control participants did not adhere to the individual 2018 WCRF/AICR Cancer Prevention Recommendation of ‘being a healthy weight’ (BMI < 25 kg/m^2^). Similar low adherence to this recommendation was observed in an American cohort study (Black Women’s Health Study)^(34)^. However, more than 60 % of breast cancer cases and controls in the European Prospective Investigation into Cancer and Nutrition cohort study adhered to the individual recommendation of ‘Be a healthy weight’^(35)^. Similar high adherence to this recommendation was observed in a Moroccan-matched case–control study (1516 cases and 1516 controls), a Spanish cohort study (*n* 10 930) and the MCC case–control study conducted in Spain (1343 breast cancer cases)^(10,36)^. In addition, this study showed concerning low levels of adherence (5 % of controls and 6 % of breast cancer cases) to the 2018 individual WCRF/AICR Cancer Prevention Recommendation to ‘be physically active’ (>150 min of physical activity per week). The case–control study of Turati *et al.* (2020) conducted in Italy and Switzerland showed higher adherence to this particular individual recommendation (30·1 % of controls and 23·5 % of breast cancer cases). Low adherence to these particular recommendations is concerning. Physical inactivity may increase the risk of being overweight or obese and being overweight or obese may increase postmenopausal breast cancer risk^(18)^.

As a result of highly skewed distributions in this study, the remaining adherence categories (meeting and partially meeting) have too little participants, to obtain sufficient statistical power, given the sample size. Thus, using predefined NCI-led cut-offs may not reflect true associations with adherence to the 2018 WCRF/AICR Cancer Prevention Recommendations and breast cancer risk in this study population. Therefore, data-driven tertiles were used to determine more evenly distributed cut-points to assess adherence to an adapted version of the 2018 WCRF/AICR Cancer Prevention Recommendations. However, results on adherence to the adapted WCRF/AICR Cancer Prevention Recommendations should be interpreted with caution as the adapted score only refers to a partially healthier lifestyle for this non-Caucasian population (while yet not adhering to the WCRF/AICR’s recommendations), as such requiring further investigation.

In this study, adherence to the overall adapted WCRF/AICR Cancer Prevention Recommendations (an adherence score >4·5–8) was low in both breast cancer case and control participants, and the vast majority of our study participants (over 80 % in both case and control participants) were not following a healthy lifestyle. Low adherence to the adapted WCRF/AICR Cancer Prevention Recommendations may be attributed to not only the income levels of this population but also the high availability of ultraprocessed foods. In South Africa, a diet rich in wholegrains, fruit and vegetables can cost up to 69 % more than a diet consisting of ultraprocessed, known to be energy-dense and nutrient-poor foods (fast foods, processed meat and sugar-sweetened drinks)^(37)^. Thus, lower-income populations may not be able to afford or prepare healthier foods as recommended by the WCRF/AICR. This is worrisome as results of this study indicated that higher adherence to the adapted WCRF/AICR Cancer Prevention Recommendations may protect against the development of breast cancer in this population.

In particular, higher adherence to the adapted individual recommendations of ‘consuming fruit and vegetables’ (>543 g/d) and ‘limit consumption of fast foods and other processed foods high in fat, refined starches or sugars’ (<20·1 % of total energy intake/d) showed strong inverse associations with breast cancer risk in this study. Similar results were observed in the case–control study of Turati *et al.* (2020), which showed that greater consumption of wholegrains, fruits and vegetables and greater limitation of fast food and other processed foods high in fat, refined starches or sugars were associated with a reduction in breast cancer risk overall (OR = 0·75, 95 % CI 0·63, 0·90 and OR = 0·63, 95 % CI 0·50, 0·80, respectively). However, when adherence was compared with the 2018 WCRF/AICR Cancer Prevention Recommendations, low adherence was noted in both breast cancer cases and controls for ‘consuming fruit and vegetables (>400 g/d)’ and ‘limit consumption of fast foods and other processed foods high in fat, refined starches or sugars (<20·1 % of total energy intake/d). The low consumption of fruit and vegetables and higher consumption of ultraprocessed foods may partially be explained by their cost, availability and convenience^(38,39)^. Due to rapid economic growth and urbanisation, the ongoing nutrition transition in South Africa contributes to more obesogenic food environments and displacement of whole foods with ultraprocessed foods^(40–43)^. Intervention is therefore required to improve accessibility, availability and affordability of healthier (nutrient-dense) foods such as fruit and vegetables to enhance healthier lifestyles of the South African population.

Only two of the adapted individual WCRF/AICR Cancer Prevention Recommendations, consuming fruit and vegetables and limit consumption of fast foods and processed foods high in added sugar, fat and refined starches, showed significant inverse associations with breast cancer risk in this study. Yet, adherence to the overall adapted WCRF/AICR Cancer Prevention Recommendations showed a strong inverse association with breast cancer risk overall, in postmenopausal women, in women with ER+ and PR+ breast cancers and in obese women. This highlights the importance of following an overall healthy lifestyle pattern, instead of adhering only individual WCRF/AICR Cancer Prevention Recommendations for cancer prevention. The WCRF/AICR Cancer Prevention Recommendations on diet, body weight and physical activity should therefore be promoted as a major tool for cancer prevention.

Interestingly, smaller waist circumferences (<92 cm) were positively associated with breast cancer risk, while having a lower BMI (<28·6 kg/m^2^) compared with having higher BMI (>34·7 kg/m^2^) did not show any significant association with breast cancer risk in our study. However, strong evidence shows that greater body weight increases postmenopausal breast cancer risk^(18)^. In this study, almost half of the breast cancer cases were diagnosed in later stages of breast cancer (stage III/IV), which could cause involuntary weight loss due to advanced cancer symptoms^(44,45)^. Thus, breast cancer cases with late-stage cancer diagnoses may have lost weight prior participation in the study and may therefore not reflect their usual weight. In addition, the way fat is distributed in the body may also influence breast cancer risk. The positive association between breast cancer risk and a smaller waist circumference should be interpreted with caution and requires further investigation.

Greater adherence to the adapted recommendation ‘limiting alcohol consumption’ showed a positive association with premenopausal breast cancer risk in this study. Yet, robust evidence shows that alcohol consumption (no identified threshold) increases both pre-and-postmenopausal breast cancer risk, especially in higher-income countries^(18)^. In this study, controls had a higher percentage of alcohol consumers (cases = 19·2 % and controls = 30·6 %) together with low quantities of alcohol (ethanol) consumption among alcohol consumers (cases = 5·4 g and controls = 4·6 g). Low alcohol intakes and consumers of alcohol, and low socio-economic status together with the small sample size may contribute to the unexpected results. The positive association between adhering to alcohol recommendations and breast cancer risk in this study should be interpreted with caution and requires further investigation.

### Limitations

The sample size of this study is rather limited (based upon sample size calculations for breast cancer overall and for stratified analysis) but provides much-needed evidence to consider in breast cancer prevention policies in South Africa. No physical examination (blood tests, cancer screening) was performed to determine whether control participants were breast cancer-free and could have been asymptomatic or undiagnosed. Dietary intake was measured over the past month when the habitual dietary intake of case participants could have changed due to illness. Seasonal variability of foods may have influenced usual dietary intakes. The nature of the study design is more prone to information bias since dietary data collection relies on the memory of participants. Also, the homogeneity of the study population for some of the individual WCRF/AICR recommendations may be considered as a potential limitation as no associations can be found when most of the people are sharing the same characteristic.

### Strengths

This study had improved statistical precision due to the population-based and matched case–control study design (analysis for overall breast cancer risk and different breast cancer types). Cases were recruited prior to any breast cancer treatment; questionnaires used to obtain data were proven to be validated and data used in the analysis were standardised. In addition, sensitivity analyses excluding under- and over-reporters and HIV-positive cases did not modify our results. This study provides quantitative evidence for assessing adherence to the 2018 WCRF/AICR Cancer Prevention Recommendations in low-and-middle-income populations where cancer research is lacking. In addition, this study included two complementary approaches to investigate associations between breast cancer risk and adherence to the 2018 WCRF/AICR Cancer Prevention Recommendations, namely the fixed cut-off approach as well as a data-driven approach in response to the very skewed distributions that were found for some of the individual WCRF/AICR Cancer Prevention Recommendation.

In conclusion, greater adherence to the adapted WCRF/AICR’s Cancer Prevention Recommendations is associated with a reduced breast cancer risk in this black urban female population. In addition, higher consumption of fruit and vegetables and reduced consumption of fast/processed foods may play a key role in reducing breast cancer risk in this urban population. Considering the very low number of participants adhering to the individual 2018 WCRF/AICR Cancer Prevention Recommendations on being at a healthy weight and being physically active, adherence to these recommendations should be encouraged to promote health. Last, in an effort to promote the overall health of all South Africans, intervention is required to improve accessibility, availability and affordability of healthier foods such as fresh fruits and vegetables in South Africa.

## Supplementary material

For supplementary material accompanying this paper visit https://doi.org/10.1017/S0007114521001598. click here to view supplementary material
