# Supplementary material for: Adherence to cancer prevention recommendations is associated with a lower breast cancer risk in black urban South African women
Source: Br J Nutr. 2021 May 14;127(6):927–38. doi: 10.1017/S0007114521001598 (PMC8908012; doi:10.1017/S0007114521001598)
Supplement: Supplementary file 1 [file S0007114521001598sup001.docx]

**Supplementary Table 1 Distribution of adherence to the individual recommendations of the 2018 WCRF/AICR Cancer Prevention Recommendations between breast cancer cases and controls, using the NCI-led standardized scoring algorithm.**

| **2018 WCRF/AICR cancer prevention recommendation** | **NCI-led standardized scoring algorithm** | | | |  |
| --- | --- | --- | --- | --- | --- |
|  | **Operationalization** | **Score contribution** | **Controls (n=396) n (%)** | **Cases**  **(n=396) n (%)** | **P-Value** |
| 1. Be a healthy weight * | BMI: 18.5 - 24.9 kg/m2 | 0.5 | 63 (15.9) | 71 (18.1) |  |
|  | BMI: 25.0 - 29.9 kg/m2 | 0.25 | 93 (23.5) | 87 (21.9) | 0.710 |
|  | BMI <18.5 or BMI ≥30.0 kg/m2 | 0 | 240 (60.6) | 238 (60.0) |  |
|  | Waist circumference: <80 cm | 0.5 | 53 (13.4) | 63 (15.9) |  |
|  | 80 cm≤ Waist circumference <88 cm | 0.25 | 61 (15.4) | 82 (20.7) | 0.056 |
|  | Waist circumference: ≥88 cm | 0 | 282 (71.2) | 251 (63.4) |  |
| 2. Be physically active | ≥150 moderate-vigorous PA min/week | 1 | 19 (4.8) | 23 (5.8) |  |
|  | >75 & <150 moderate-vigorous PA min/week | 0.5 | 72 (18.2) | 101 (25.5) | 0.047 |
|  | ≤75 moderate-vigorous PA min/week | 0 | 305 (77.0) | 272 (68.7) |  |
| 3. Eat a diet rich in wholegrains, vegetables, fruit and beans † | Fruits and vegetables ≥400 g/day | 0.5 | 165 (41.7) | 124 (31.3) |  |
|  | 200 g ≤ Fruits and vegetables <400 g/day | 0.25 | 82 (20.7) | 102 (25.8) | 0.009 |
|  | Fruits and vegetables <200 g/day | 0 | 149 (37.6) | 170 (42.9) |  |
|  | Dietary fibre ≥30 g/day | 0.5 | 104 (26.3) | 102 (25.8) |  |
|  | 15 g/day ≤ Dietary fibre <30 g/day | 0.25 | 237 (59.8) | 241 (60.9) | 0.956 |
|  | Dietary fibre <15 g/day | 0 | 55 (13.9) | 53 (13.4) |  |
| 4. Limit consumption of “fast foods” and other processed foods high in fat, refined starches or sugars | Total kJ from UPFs ≤20.1% of TEI | 1 | 133 (33.6) | 152 (38.4) |  |
|  | 20.1% < Total kJ from UPFs ≥50.1% of TEI | 0.5 | 129 (32.6) | 156 (39.4) | 0.001 |
|  | Total kJ from UPFs >50.1% of TEI | 0 | 134 (33.8) | 88 (22.2) |  |
| 5. Limit consumption of red & processed meat | Red meat <500 g/week and processed meat intake <21 g/week | 1 | 77 (19.4) | 101 (25.5) |  |
|  | Red meat <500 g/week and processed meat intake ≥21 g/week & ≤100 g/week | 0.5 | 89 (22.5) | 100 (25.3) | 0.034 |
|  | Red and processed meat >500 g/week or processed meat intake ≥100 g/week | 0 | 230 (58.1) | 195 (49.2) |  |
| 6. Limit consumption of sugar-sweetened drinks | Sugary drinks: 0 g/day | 1 | 87 (22.0) | 89 (22.5) |  |
|  | 0 g/day < Sugary drinks <250 g/day | 0.5 | 199 (50.3) | 206 (52.0) | 0.768 |
|  | Sugary drinks ≥250 g/day | 0 | 110 (27.7) | 101 (25.5) |  |
| 7. Limit alcohol consumption | Ethanol intake 0g/day | 1 | 321 (81.1) | 350 (88.3) |  |
|  | 0 g/day< Ethanol & <14g/day | 0.5 | 60 (15.1) | 37 (9.4) | 0.001 |
|  | Ethanol intake ≥14g/day | 0 | 15 (3.8) | 9 (2.3) |  |
| 8. Don't rely on supplements for cancer prevention | Not applicable to this population |  |  |  |  |
| 9. For mothers: breastfeed your baby, if you can ‡ | Cumulative breastfeeding ≥6 months | 1 | 259 (74.2) | 242 (71.4) |  |
|  | 0< Cumulative breastfeeding <6 months | 0.5 | 39 (11.2) | 50 (14.7) | 0.347 |
|  | Never breastfed | 0 | 51 (14.6) | 47 (13.9) |  |
| 10. After a cancer diagnosis: follow our recommendations, if you can | Not applicable to this population |  |  |  |  |

WCRF; World Cancer Research Fund, AICR; American Institute for Cancer Research, PA; physical activity, ED; energy density,
* Adherence to the recommendation “Be a healthy weight” is measured by two subgroups, BMI and waist circumference.

† Adherence to the recommendation “Eat a diet rich in wholegrains, vegetables, fruit and beans” is measured by two subgroups, fruit and vegetable intake and daily fibre intake.

‡ Recommendation only applied to parous women (case n=382; controls n=377).

| **Supplementary Table 2 Associations between the adherence to the overall 2018 WCRF/AICR Cancer Prevention Recommendations using the NCI-led standardized scoring algorithm and breast cancer risk.** | | | | | |
| --- | --- | --- | --- | --- | --- |
|  | Adherence score | **Controls n (%)** | **Cases n (%)** | **Model 1** | **Adjusted Model 2** |
| **Overall (cases=396; controls=396)** | Per 1-point increment |  |  | 0.81 (0.70-0.94) | 0.79 (0.68-0.93) |
|  | P value |  |  | 0.005 | 0.005 |
|  | ≤3.5 | 154 (38.8) | 180 (45.5) | 1 |  |
|  | >3. 5 & ≤4.5 | 142 (35.9) | 139 (35.1) | 0.82 (0.59-1.12) | 0.85 (0.61-1.19) |
|  | >4.5 | 100 (25.3) | 77 (19.4) | 0.65 (0.44-0.94) | 0.61 (0.41-0.93) |
|  | P-trend |  |  | 0.023 | 0.021 |
| ***Premenopausal (n=287)  (cases n=133; controls n=134)** | Per 1-point increment |  |  | 0.75 (0.56-1.00) | 0.73 (0.56-0.96) |
|  | P value |  |  | 0.055 | 0.027 |
|  | ≤3.5 | 42 (31.3) | 58 (43.6) | 1 | 1 |
|  | >3. 5 & ≤4.5 | 54 (40.3) | 50 (37.6) | 0.64 (0.33-1.24) | 0.65 (0.37-1.16) |
|  | >4.5 | 38 (28.4) | 25 (18.8) | 0.52 (0.25-1.05) | 0.46 (0.23-0.90) |
|  | P-trend |  |  | 0.071 | 0.023 |
| ***Postmenopausal (n=505)  (cases n=248; controls n=257)** | Per 1-point increment |  |  | 0.86 (0.71-1.04) | 0.84 (0.70-1.010 |
|  | P value |  |  | 0.113 | 0.068 |
|  | ≤3.5 | 111 (43.1) | 115 (46.4) | 1 | 1 |
|  | >3. 5 & ≤4.5 | 86 (33.5) | 87 (35.1) | 1.00 (0.67-1.50) | 0.98 (0.65-1.50) |
|  | >4.5 | 60 (23.4) | 46 (18.5) | 0.78 (0.48-1.30) | 0.72 (0.44-1.19) |
|  | p-trend |  |  | 0.350 | 0.209 |
| **ER+ (n= 298)** | Per 1-point increment |  |  | 0.69 (0.50-0.97) | 0.69 (0.48-0.98) |
|  | P value |  |  | 0.032 | 0.051 |
|  | ≤3.5 | - | 121 (40.6) | 1 | 1 |
|  | >3. 5 & ≤4.5 | - | 97 (32.6) | 0.66 (0.33-1.33) | 0.77 (0.36-1.67) |
|  | >4.5 | - | 80 (26.8) | 0.41 (0.17-1.04) | 0.40 (0.15-1.09) |
|  | P-trend |  |  | 0.601 | 0.075 |
| **PR+ (n=263)** | Per 1-point increment |  |  | 0.57 (0.31-0.89) | 0.63 (0.45-0.78) |
|  | P value |  |  | <0.001 | 0.011 |
|  | ≤3.5 | - | 109 (41.4) | 1 | 1 |
|  | >3. 5 & ≤4.5 | - | 91 (34.6) | 0.49 (0.27-0.88) | 0.60 (0.31-1.18) |
|  | >4.5 | - | 63 (24.0) | 0.31 (0.14-0.68) | 0.35 (0.14-0.87) |
|  | P-trend |  |  | 0.004 | 0.024 |
| ***†Obese (n=466) Cases=231 Controls=235** | Per 1-point increment |  |  | 0.79 (0.66-0.95) | 0.76 (0.62-0.93) |
|  | P value |  |  | 0.013 | 0.009 |
|  | ≤3.5 | 111 (47.2) | 123 (53.3) | 1 | 1 |
|  | >3. 5 & ≤4.5 | 85 (36.2) | 77 (33.3) | 0.86 (0.61-1.23) | 0.84 (0.62-2.01) |
|  | >4.5 | 39 (16.6) | 31 (13.4) | 0.71 (0.47-1.08) | 0.65 (0.38-1.20) |
|  | P-trend |  |  | 0.108 | 0.832 |

ER+, oestrogen receptor positive; PR+, Progesterone receptor positive

Model 1: crude model

Model 2: Adjusted for total energy intake, ethnicity, level of education, level of income and menopausal status (not adjusted for menopausal status when stratified by menopausal status).

*Unconditional logistic regression as case and control participants were not matched on menopausal status nor on obesity.
† Obesity defined as BMI ≥30 kg/m^2^

**Supplementary Table 3 Associations between the adherence to individual recommendations of the 2018 WCRF/AICR Cancer Prevention Recommendation, using the NCI-led standardized cut-points and breast cancer risk**

| **WCRF/AICR cancer prevention recommendation** |  | **Overall#**  **(cases=396; controls=396)** | ***‡Premenopausal**  **(cases=133; controls=134)** | ***‡Postmenopausal**  **(cases=248; controls=257)** | **†ER + (n=312)** | **†PR+ (281)** |
| --- | --- | --- | --- | --- | --- | --- |
|  |  | **OR (95% CI)** | **OR (95% CI)** | **OR (95% CI)** | **OR (95% CI)** | **OR (95% CI)** |
| **Be a healthy weight (1)**  BMI | 0 | 1 | 1 | 1 | 1 | 1 |
|  | 0.5 | 1.00 (0.70-1.42) | 0.83 (0.44-1.58) | 0.98 (0.61-1.57) | 0.88 (0.59-1.30) | 1.02 (0.49-2.17) |
|  | 1 | 1.23 (0.83-1.83) | 1.50 (0.75-3.02) | 1.14 (0.67-1.95) | 1.34 (0.87-2.07) | 1.16 (0.50-2.79) |
|  | P-trend | 0.856 | 0.383 | 0.865 | 0.183 | 0.747 |
| **Be a healthy weight (1)**  Waist circumference | 0 | 1 | 1 | 1 | 1 | 1 |
|  | 0.5 | 1.62 (1.08-2.41) | 1.02 (0.55-1.92) | 1.88 (1.15-3.07) | 2.30 (0.89-5.88) | 2.72 (1.22-6.10) |
|  | 1 | 1.31 (0.84-2.04) | 1.04 (0.56-1.88) | 1.4 (0.77-2.60) | 0.96 (0.34-2.72) | 1.36 (0.52-3.54) |
|  | P-trend | 0.221 | 0.921 | 0.257 | 0.949 | 0.526 |
| **Be physically active** | 0 | 1 | 1 | 1 | 1 | 1 |
|  | 0.5 | 0.60 (0.26-1.41) | 0.94 (0.20-4.44) | 0.46 (0.14-1.54) | 0.66 (0.26-1.68) | 0.40 (0.07-2.44) |
|  | 1 | 1.11 (0.62-1.98) | 1.31 (0.50-3.41) | 1.19 (0.52-2.74) | 1.17 (0.61-2.27) | 0.63 (0.19-2.09) |
|  | P-trend | 0.171 | 0.766 | 0.114 | 0.611 | 0.453 |
| **Eat a diet rich in wholegrains, vegetables, fruit and beans** **(2)**  Fruit and vegetable intake | 0 | 1 | 1 | 1 | 1 | 1 |
|  | 0.5 | 1.03 (0.37-1.04) | 0.78 (0.40-1.53) | 1.30 (0.81-2.09) | 1.12 (0.70-1.78) | 1.38 (0.62-3.01) |
|  | 1 | 0.57 (0.40-0.83) | 0.67 (0.38-1.19) | 0.62 (0.41-0.94) | 0.56 (0.37-0.85) | 0.76 (0.36-1.62) |
|  | P-trend | 0.003 | 0.179 | 0.026 | 0.007 | 0.481 |
| **Eat a diet rich in wholegrains, vegetables, fruit and beans** **(2)**  Daily fiber intake | 0 | 1 | 1 | 1 | 1 | 1 |
|  | 0.5 | 0.85 (0.54-1.35) | 0.62 (0.24-1.63) | 0.94 (0.56-1.58) | 0.68 (0.22-2.16) | 0.60 (0.24-1.54) |
|  | 1 | 0.82 (0.46-1.49) | 0.39 (0.13-1.18) | 1.22 (0.61-2.42) | 1.08 (0.29-4.04) | 0.77 (0.23-2.54) |
|  | P-trend | 0.530 | 0.095 | 0.562 | 0.909 | 0.672 |
| **Limit fast foods** | 0 | 1 | 1 | 1 | 1 | 1 |
|  | 0.5 | 0.84 (0.51-1.11) | 0.80 (0.45-1.79) | 0.97 (0.68-1.49) | 0.79 (0.36-1.71) | 0.64 (0.49-1.55) |
|  | 1 | 0.64 (0.38-0.92) | 0.65 (0.44-1.1) | 0.64 (0.41-1.09) | 0.49 (0.17-1.13) | 0.53 (0.33-1.45) |
|  | P-trend | 0.029 | 0.092 | 0.099 | 0.081 | 0.221 |
| **Limit red & processed meat** | 0 | 1 | 1 | 1 | 1 | 1 |
|  | 0.5 | 0.80 (0.52-1.23) | 1.00 (0.44-2.28) | 0.69 (0.41-1.18) | 0.75 (0.45-1.23) | 0.58 (0.26-1.34) |
|  | 1 | 0.58 (0.37-1.04) | 0.69 (0.34-1.43) | 0.51 (0.32-1.03) | 0.53 (0.34-1.02 | 0.32 (0.13-1.00) |
|  | P-trend | 0.319 | 0.331 | 0.177 | 0.257 | 0.208 |
| **Limit sugary drinks** | 0 | 1 | 1 | 1 | 1 | 1 |
|  | 0.5 | 1.01 (0.69-1.48) | 1.13 (0.59-2.14) | 0.96 (0.55-1.66) | 1.03 (0.67-1.58) | 0.67 (0.31-1.41) |
|  | 1 | 0.94 (0.60-1.48) | 1.08 (0.45-2.61) | 0.83 (0.45-1.53) | 0.80 (0.47-1.37) | 0.85 (0.37-1.98) |
|  | P-trend | 0.805 | 0.568 | 0.786 | 0.539 | 0.706 |
| **Limit alcohol consumption** | 0 | 1 | 1 | 1 | 1 | 1 |
|  | 0.5 | 0.19 (0.03-1.37) | 0.35 (0.07-1.86) | 1.03 (0.46-2.25) | 0.19 (0.28-1.37) | 0.61 (0.15-2.46) |
|  | 1 | 1.49 (0.56-3.92) | 2.33 (0.89-4.31) | 1.56 (0.79-3.08) | 1.59 (0.56-3.62) | 2.04 (0.92-4.52) |
|  | P-trend | 0.413 | 0.220 | 0.199 | 0.456 | 0.080 |
| **Breastfeed your baby, if you can** | 0 | 1 | 1 | 1 | 1 | 1 |
|  | 0.5 | 1.32 (0.77-2.24) | 2.60 (0.81-8.37) | 1.02 (0.51-2.05) | 1.08 (0.60-1.96) | 1.97 (0.63-6.14) |
|  | 1 | 0.80 (0.57-1.12) | 0.75 (0.40-1.41) | 0.80 (0.50-1.28) | 0.92 (0.63-1.34) | 0.73 (0.37-1.46) |
|  | P-trend | 0.085 | 0.137 | 0.493 | 0.787 | 0.380 |

ER+, oestrogen receptor positive; PR+, Progesterone receptor positive
0 indicates the lowest adherence to the specific recommendation
#Overall: Adjusted for Adjusted for total energy intake, individual income/month, ethnicity, level of education, physical activity, waist circumference, alcohol intake, (unless the variable was part of the recommendation under investigation) and menopausal status (not when stratified by menopausal status)
* Stratified by menopausal status or obesity and using unconditional logistic regression.
‡20 missing values for menopausal status (15 cases and 5 controls).
† Stratified by oestrogen receptor status or Progesterone receptor status
(1) Adherence to the recommendation “Be a healthy weight” is measured by two subgroups, BMI and waist circumference.

(2) Adherence to the recommendation “Eat a diet rich in wholegrains, vegetables, fruit and beans” is measured by two subgroups, fruit and vegetable intake and daily fiber intake.
